# The complete chloroplast genome sequence of *Iris tectorum*

**DOI:** 10.1080/23802359.2021.1895002

**Published:** 2021-11-03

**Authors:** Jinglu Feng, Yunjia Pan, Yulin Lin, Hui Yao

**Affiliations:** a Institute of Medicinal Plant Development, Chinese Academy of Medical Sciences and Peking Union Medical College, Beijing, China; b Engineering Research Center of Chinese Medicine Resource, Ministry of Education, Beijing, China

**Keywords:** *Iris tectorum*, chloroplast genome, phylogenetic analysis

## Abstract

The complete chloroplast genome sequence of *Iris tectorum* Maximowicz, assembled with Illumina NovaSeq 6000 system platform sequencing data, was reported. The total length of the chloroplast genome of *I. tectorum* is 153,253 bp and its GC content is 37.89%. The complete chloroplast genome has four distinct parts a large single copy region (82,833 bp), a small single copy region (18,562 bp), and a pair of inverted repeats (25,929 bp). The chloroplast genome includes 86 protein-coding genes, 8 rRNAs and 38 tRNAs genes. A phylogenetic tree showed that *I. tectorum* is close to *Iris missouriensis*.

*Iris tectorum* is a perennial plant, with thick creeping rhizomes and bluish violet flowers. It lives in places beside water, such as in forest margins, sunny banks, meadows and damp places, at altitude of 500–3500 m. *I. tectorum* belongs to the Iridaceae family of the Asparagales order (Wu et al. 2013; Chase et al. 2016), and its rhizome is a traditional Chinese medicine named Iridis Tectori Rhizoma. This medicine is used to clear heat to detoxifyand to throat-swelling diseases (Zhao 1985; National Pharmacopoeia Committee 2020). It has anti-inflammatory, expectorant, and anti-virus effects (Xu et al. 2018). Studies on its genome are limited, so the chloroplast genome was examined and phylogenetic analysis of *I. tectorum* was conducted.

Fresh leaves of *I. tectorum* were collected from Medicinal Plant Garden of Institute of Medicinal Plant Development (IMPLAD), Beijing (40°2′5″N, 116°16′14″E). The specimen was deposited in the herbarium of IMPLAD (Jinglu Feng 472948673@qq.com) under the voucher number Feng 001. Its total genomic DNA was extracted using QIAGEN DNeasy Plant Mini Kit and sequenced on an Illumina NovaSeq 6000 system platform. Clean data were assembled into a complete chloroplast genome by NOVOPlasty (https://github.com/ndierckx/NOVOPlasty) and repaired by GapCloser (v1.12, http://soap.genomics.org.cn/soapdenovo.html). The protein-coding genes, rRNA genes, and tRNA genes were annotated by using GeSeq (Tillich et al. 2017, https://chlorobox.mpimp-golm.mpg.de/geseq.html), coupled with manual correction.Phylogenetic analysis was constructed based on the 14 complete chloroplast genome sequences. Maximum likelihood (ML) analysis was conducted with a bootstrap of 1000 repetitions based on the TVM + F+R3 nucleotide substitution model using IQ-TREE software (http://www.iqtree.org/). This adopted best-fit model was determined by ModelFinder.

The total length of the chloroplast genome of *I. tectorum* is 153,253 bp (GenBank accession number: MW201731), and its GC content is 37.89%. Four parts of the complete genome, namely, a large single copy region, a small single copy region and a pair of inverted repeats are 82,833, 18,562 and 25,929 bp, respectively. In the genome, 86 protein-coding genes, 8 rRNA genes and 38 tRNA genes were annotated by using GeSeq.

A 1000-bootstrap-replicate ML tree (Figure 1) was used for the phylogenetic analysis of the complete chloroplast genome of *I. tectorum* and 13 species, belonging to the Asparagales order. The tree demonstrates that *I. tectorum* is close to *Iris missouriensis*.

**Figure 1.**
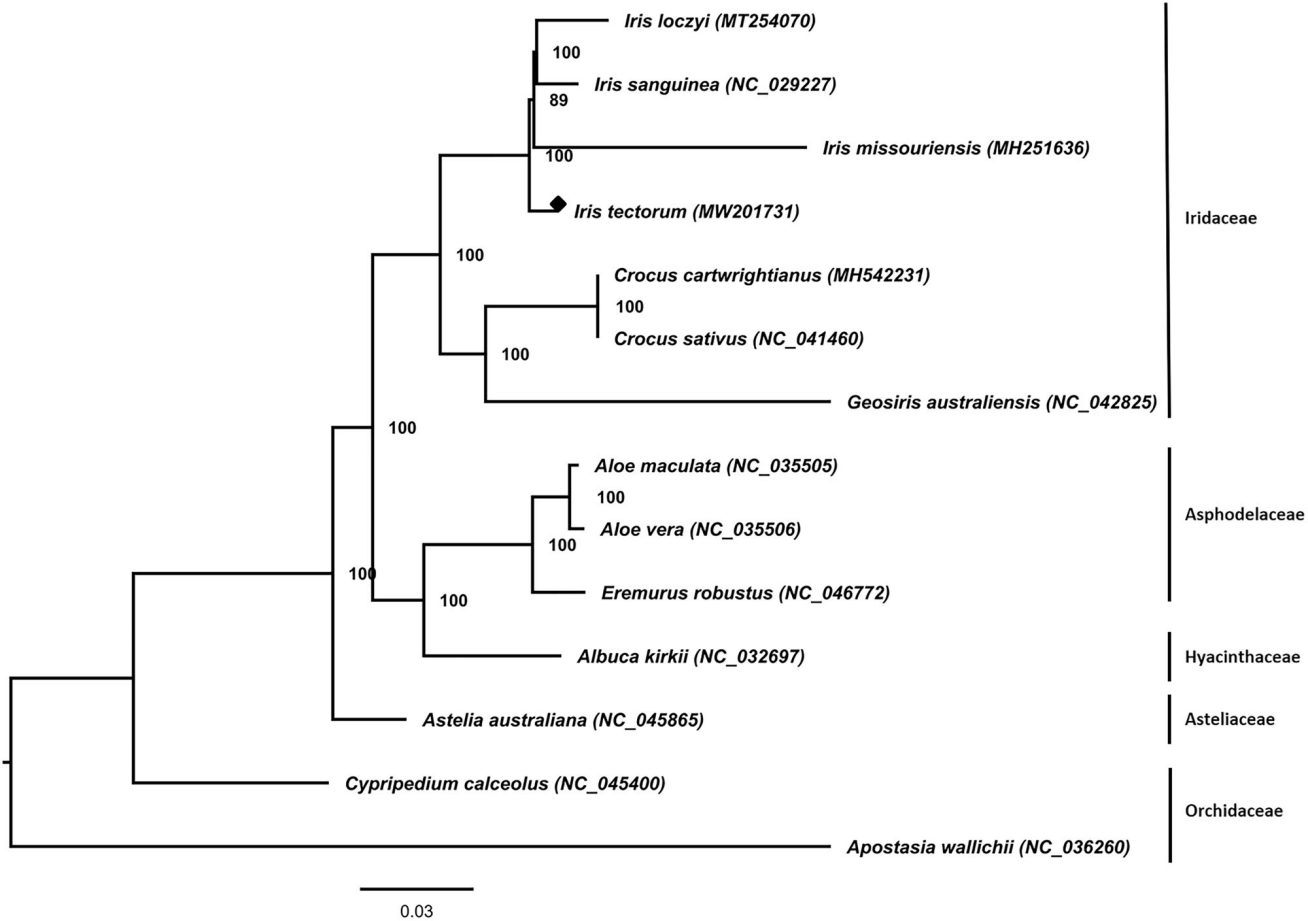
Phylogenetic tree of *Iris tetcorum* and 13 species in the order Asparagales using maximum likelihood (ML) analyses based on the complete chloroplast genome sequences. The numbers at nodes of the phylogenetic tree show the bootstrap support values.

## Data Availability

The data that support the findings of this study are openly available in NCBI athttps://www.ncbi.nlm.nih.gov/nuccore/MW201731 The genome sequence data that support the findings of this study are openly available in GenBank of NCBI at [https://www.ncbi.nlm.nih.gov] (https://www.ncbi.nlm.nih.gov/) under the accession No.SAMN17169715. The associated BioProject, SRA, and Bio-Sample numbers are PRJNA688136, SRR13311445, and SAMN17169715 respectively.
